# Diagnostic Evaluation of Des-Gamma-Carboxy Prothrombin versus α-Fetoprotein for Hepatitis B Virus-Related Hepatocellular Carcinoma in China: A Large-Scale, Multicentre Study

**DOI:** 10.1371/journal.pone.0153227

**Published:** 2016-04-12

**Authors:** Jun Ji, Hao Wang, Yan Li, Lei Zheng, Yuepeng Yin, Zhenzhen Zou, Feiguo Zhou, Weiping Zhou, Feng Shen, Chunfang Gao

**Affiliations:** 1 Department of Laboratory Medicine, Eastern Hepatobiliary Surgery Hospital, Second Military Medical University, Shanghai, China; 2 Department of Laboratory Medicine, Changzheng Hospital, Second Military Medical University, Shanghai, China; 3 Department of Laboratory Medicine, Renmin Hospital, Wuhan University, Hubei, China; 4 Department of Laboratory Medicine, Nanfang Hospital, Southern Medical University, Guandong, China; 5 Department of Liver Surgery, Eastern Hepatobiliary Surgery Hospital, Second Military Medical University, Shanghai, China; University of Modena & Reggio Emilia, ITALY

## Abstract

An efficient serum marker for hepatocellular carcinoma (HCC) is currently lacking and requires intensive exploration. We aimed to evaluate the performance of des-gamma-carboxy prothrombin (DCP) for identifying hepatitis B virus-related HCC in a large, multicentre study in China. A total of 1034 subjects in three cohorts (A, B, and C) including HCC and various non-HCC controls were enrolled from 4 academic medical centers in China from January 2011 to February 2014. Blind parallel detections were conducted for DCP and AFP. The area under the receiver operating characteristic curve (AUC) was used to evaluate the diagnostic efficacies. In cohort A, which comprised 521 subjects, including patients with HCC, liver metastasis, liver cirrhosis (LC), and liver hemangiomas as well as healthy controls (HCs), the accuracy of DCP for distinguishing HCC from various controls was 6.2–9.7% higher than that of AFP. In cohort B, which comprised 447 subjects, including patients with HCC, LC, and chronic hepatitis B as well as HC, the accuracy of DCP was further elevated (12.3–20.67% higher than that of AFP). The superiority of DCP to AFP was more profound in the surveillance of early HCC [AUC 0.837 (95% CI: 0.771–0.903) vs. 0.650 (0.555–0.745)] and AFP-negative HCC [AUC: 0.856 (0.798–0.914)] and in discriminating HCC from LC (accuracy: 92.9% vs.64.71%). Higher DCP levels were associated with worse clinical behaviors and shorter disease-free survival. DCP not only is complementary to AFP in identifying AFP-negative HCC and in excluding AFP-positive non-HCC (liver cirrhosis), but also demonstrates improved performance in HCC surveillance, early diagnosis, treatment response and recurrence monitoring in the HBV-related population.

## Introduction

Hepatocellular carcinoma (HCC) is the most common type of primary liver cancer, accounting for 84% of all liver cancer cases [1]. Although liver resection for early HCC could improve 5-year survival to 60–70% [2], the overall 5-year survival rate is less than 40%. This worse outcome is due partly to the lack of an effective method for timely diagnosis, which leads to only 30–40% of HCC being suitable for potentially curative treatments at the time of diagnosis [3,4].

Alpha-fetoprotein (AFP) was first introduced as a serological marker for HCC in the1960s [5]. However, many patients with non-malignant chronic liver diseases, such as 15–58% of those with chronic hepatitis and 11–47% of those with liver cirrhosis [6, 7], have elevated AFP concentrations. In contrast, approximately 30–40% of HCCs show normal serum AFP levels. The American Association for the Study of Liver Diseases (AASLD) Practice Guidelines Committee recommended that ultrasound (US) exam alone be used for HCC surveillance due to the poor sensitivity and specificity of AFP in detecting early-stage HCC [8].

Des-γ-carboxy prothrombin (DCP), also known as the protein induced by vitamin K deficiency or antagonist-II (PIVKA-II), was first reported in 1984 [9]. Several studies have demonstrated that DCP is a useful marker for HCC, and higher DCP levels indicated worse clinical features of HCC [10,11,12].

The mechanisms of carcinogenesis for patients with HCC differ [13,14], and prominent geographic distribution differences in the causes of HCC exist worldwide [15]. Hepatitis B virus (HBV) and hepatitis C virus (HCV) infections are the major risk factors for HCC. Several studies have indicated that AFP and DCP values in HBV-related HCC differ from values of HCV-related HCC, which might be related to their different clinical manifestations and mechanisms of carcinogenesis [13,14,16,17].

Thus, although serum DCP has been revealed as a useful diagnostic and prognostic marker for HCC, the majority of large-scale studies were performed in patients with mainly HCV-related etiology [18,19,20,21,22]. Only relatively recently have a few studies appeared that explored the significance of DCP in identifying HBV-related HCC [23,24]. Here, we designed a large-scale, multi-centre validation study to evaluate the diagnostic performance of DCP in China. The temporal change of DCP (before and after curative hepatectomy) and its prognostic prediction value were also assessed.

## Materials and Methods

### Ethics Statement

Written informed consent was obtained from all study participants, and the study protocols were approved by the Chinese Ethics Committees of Human Resources, Eastern Hepatobiliary Surgery Hospital (EHBH) and Changzheng Hospital (CZH), Second Military Medical University, Renmin Hospital (RMH) of Wuhan University, and Nanfang Hospital (NFH) of Southern Medical University.

### Study population

**Fig 1:**
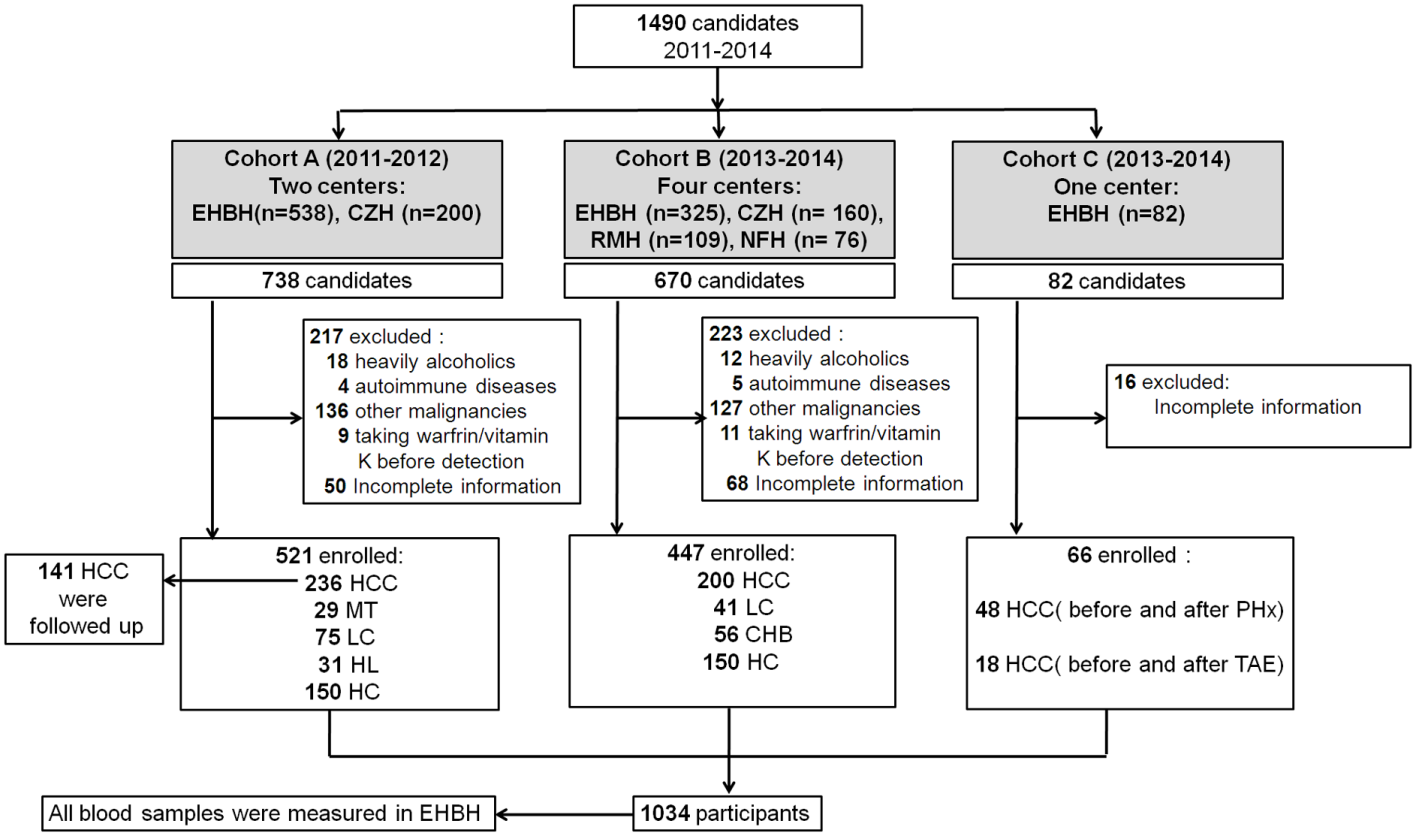
Study profile. TAE, transcatheter arterial embolization; AFP, α-fetoprotein; HCC, hepatocellular carcinoma; MT, liver metastasis; LC, liver cirrhosis; HL, hemangiomas of the liver; CHB, chronic hepatitis B virus infection; HC, healthy control; EHBH, Eastern Hepatobiliary Hospital of Second Military Medical University; CZH, Changzheng Hospital of Second Military Medical University; RMH, Renmin Hospital of Wuhan University; NFH, Nanfang Hospital of Southern Medical University.

As described in a flow chart (Fig 1),a total of 1034 patients were enrolled, of whom521 were in the cohort for differential diagnosis (cohort A), 447 were in the cohort for high-risk population surveillance (cohort B), and 66 were in the treatment-monitoring cohort (cohort C).

Cohort A comprised subjects with HCC, liver metastasis (MT), liver cirrhosis (LC), and hemangiomas of liver (HL) as well as healthy controls (HCs) who were recruited from Shanghai EHBH and Shanghai CZH from January 2011 to December 2012.

Cohort B comprised subjects with HCC, chronic hepatitis B (CHB), and LC and HCs who were recruited from EHBH, CZH, and RMH of Wuhan University in Hubei Province and from NFH of Southern Medical University in Guangdong Province from January 2013 to February 2014.

Another independent treatment-monitoring cohort comprising 66 cases of HCC [48 cases of HCC treated by curative partial hepatectomy and 18 cases by transcatheter arterial embolization (TAE)] was enrolled for before-and-after treatment comparison from EHBH. A total of 141 patients in cohort A were followed up for 26 months (median, 15.8 months) after hepatectomy, from June 2011 to July 2014, according to clinical availability.

### Definitions and Exclusion Criteria

The diagnosis of HCC was made by abdominal ultrasonography, dynamic computed tomography (CT) scanning or MRI characteristics and AFP and was confirmed by histopathology. Tumor stage was defined according to the TNM (tumor node metastasis) criteria [25]. MT and hemangiomas of liver (HL) were defined on the basis of ultrasound, CT, or MRI exam and were confirmed by histopathology. The diagnosis of CHB included the presence of HBsAg for 6 months prior, HBV DNA concentrations higher than 10^3^ IU/ml, and elevated concentrations of serum alanine aminotransferase, according to the guidelines for prevention and treatment of chronic HBV infection [26]. The diagnosis of LC was based on the histopathology of a liver biopsy or clinical, laboratory, and imaging evidence when possible. Patients with cirrhosis who had elevated AFP concentrations were required to have undergone imaging by multiple methods (ultrasonography, CT, or MRI) and to have had no evidence of a hepatic mass for at least 3 months before enrollment. HCs had routine healthy exams with normal laboratory liver/kidney function, no history of liver diseases, no viral hepatitis, and no malignant diseases.

Exclusion: Subjects who (1) were heavy alcoholics (more than 80 g of ethanol daily), (2) suffered from cholestatic autoimmune diseases, (3) were taking vitamin K or warfarin before DCP measurement, or (4) had evidence of other malignancies were excluded from this study. Because alcohol intake, obstructive jaundice, vitamin K deficiency, or taking warfarin might induce aberrant increases in serum DCP.

### Blood sample testing

Peripheral blood samples were collected at the time of diagnosis and before treatment. To assess whether levels of DCP and AFP were changed after radical partial hepatectomy or palliative therapy (TAE) for HCC, 66 pairs (before and mean 40 days after treatment) of HCC sera samples were collected. Centralized assays were performed at EHBH in a blind manner. AFP and markers of HBV infection (HBsAg, HBeAg, etc.) were measured by the electrochemiluminescence immunoassay (ECLIA) (Roche E170 Analyzer, Roche, Tokyo, Japan). DCP was determined by the chemiluminescence enzyme immunoassay (CLEIA) (LUMIPULSE G1200, Fujirebio, Tokyo, Japan). The other related biochemical parameters were detected using standard methods and matched reagents (Hitachi 7600 Analyzer, Hitachi, Tokyo, Japan; Wako diagnostic reagents, Wako Pure Chemical Industries Ltd., Osaka, Japan).

### Statistical analysis

SPSS 16.0 for Windows statistical software (SPSS Inc.) was used for statistical analyses. We compared DCP and AFP levels before and after treatment with the independent samples *t* test and the paired *t* test. Pearson’s χ^2^ test or Fisher’s exact test was constructed to analyze correlations between DCP and clinicopathological characteristics. The Mann-Whitney U test was used to test differences between two independent groups (continuous variables and non-parametric analyses). We assessed sensitivity, specificity, and respective areas under the curves (AUCs) with 95% CI by receiver operating characteristic (ROC) curves. The optimum cutoff value for diagnosis was investigated by maximizing the sum of sensitivity and specificity.

Survival curves were calculated using the Kaplan-Meier method, and the difference was determined using a log-rank test. The Cox proportional hazards model was used to identify the independent risk factors significantly associated with survival. All reported *P* values were 2-tailed, and *P*<0.05 was considered statistically significant.

## Results

### Clinicopathological features and DCP levels of enrolled subjects

The demographic characteristics of participants enrolled in Cohort A and B are summarized in S1 Table. There was a male predominance in HCC, MT, and CHB (*P*<0.0001). The majority of HCC and non-HCC disease controls (DCs, composed of HL, MT and LC in cohort A; CHB and LC in cohort B) had a current HBV infection background. In cohort A, HBV infection was detected in 214/236 (90.7%) cases of HCC and 45/75 (60%) cases of LC; in cohort B, 89% (178/200) of HCC cases and 78% (32/41) of LC cases had HBV infection.

Levels of DCP and AFP were successfully measured for all 1034 samples. Some HCC samples with DCP and/or AFP over the detection limits (AFP >1210 ng/ml, DCP >75 000 mAU/m) were diluted and further quantified according to the manufacturers’ instructions. In the differential diagnosis of patients in cohort A, DCP concentrations were significantly higher in HCC than in all non-HCC controls (median 490 mAU/ml, range 7–333568 mAU/ml; *P*<0.0001; S1A Fig, S1 Table). Elevation was also seen in MT and LC (*P*<0.0001, S1A Fig, S1 Table). Meanwhile, as expected, the median concentration of AFP was increased in HCC compared with all non-HCC controls (*P*<0.0001), and significant increases were also seen in patients with LC (*P*<0.0001, S1C Fig, S1 Table).

Cohort B showed the same trend, indicating that DCP was higher in HCC than in all non-HCC controls (median 774 mAU/ml, range 8–4146590 mAU/ml; *P*<0.0001; S1B Fig, S1 Table), and significant increases were also seen in LC (*P*<0.0001, S1B Fig, S1 Table). DCP did not differ between CHB and healthy controls, where as AFP was increased in CHB compared with healthy controls (*P*<0.0001, S1D Fig, S1 Table).

### The general diagnostic performance of DCP

**Fig 2:**
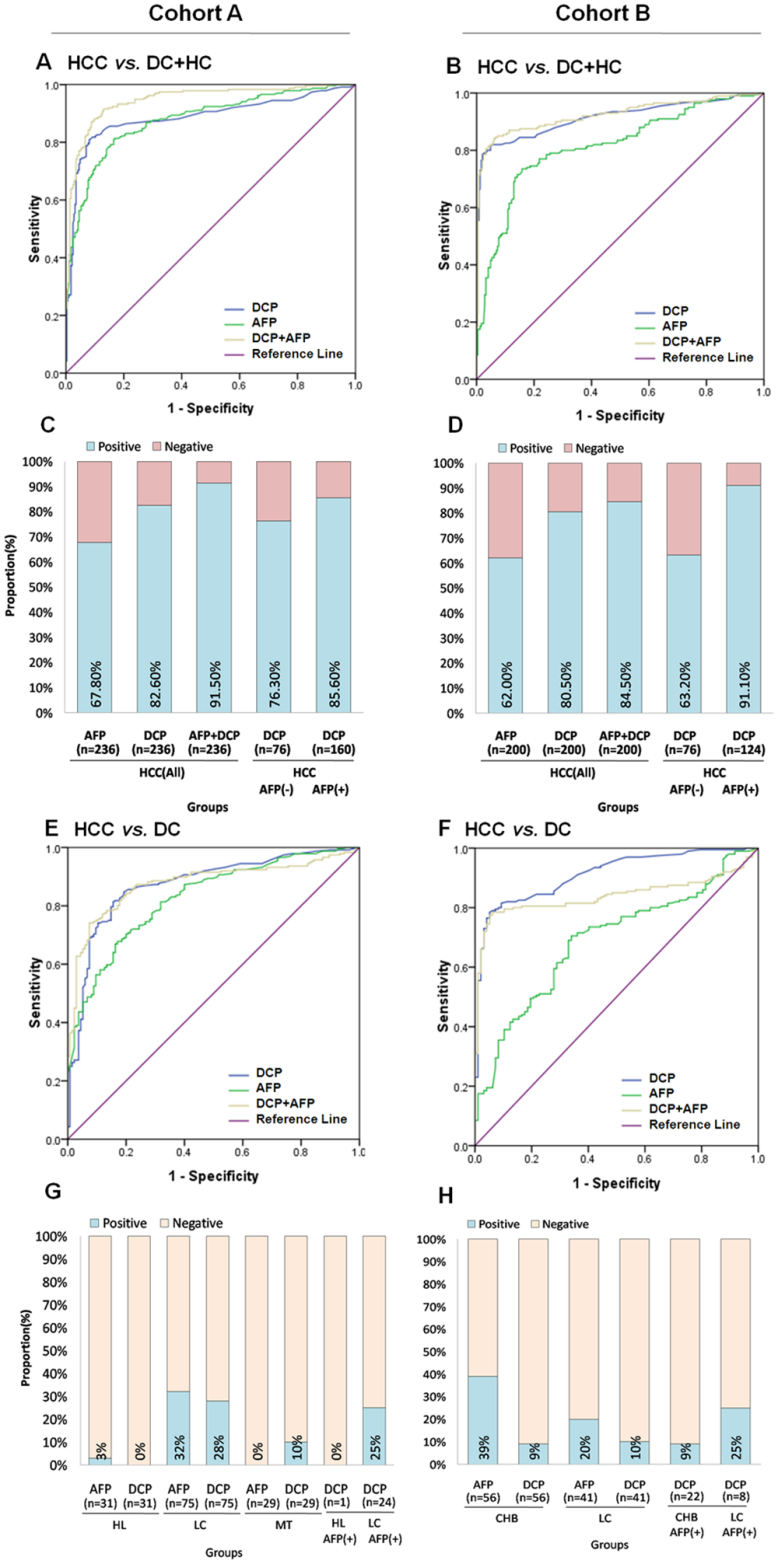
Diagnostic outcomes of DCP for HCC. (A) ROC curve for DCP, AFP, or both, for HCC versus all controls in cohort A. (B) ROC curve for DCP, AFP, or both, for HCC versus all controls in cohort B. (C) The rate of positive results for AFP, DCP, or both, in all patients with HCC and for DCP by AFP status in cohort A. (D) The rate of positive results for AFP, DCP, or both, in all patients with HCC and for DCP by AFP status in cohort B. (E) ROC curve for DCP, AFP, or both, for HCC versus disease controls in cohort A. (F) ROC curve for DCP, AFP, or both, for HCC versus disease controls in cohort B. (G) The rate of positive results of AFP and DCP for patients with hemangiomas of liver (HL), liver metastasis (MT) or cirrhosis (LC) and of DCP by AFP-positive status in cohort A. (H) The rate of positive results of AFP and DCP for chronic hepatitis B (CHB) or cirrhosis (LC) and of DCP by AFP-positive status in cohort B. ROC, receiver operating characteristics; HCC, hepatocellular carcinoma; DC, disease control; HC, healthy control; HL, hemangiomas of liver; LC, liver cirrhosis; MT, liver metastasis; CHB, chronic hepatitis B.

The accuracy and predictive values for DCP and AFP are shown in Table 1. In cohort A, the AUC of DCP for distinguishing HCC from all controls was 0.886 (95% CI 0.855–0.917, sensitivity 82.63%, specificity 89.12%) at a cutoff value of 40 mAU/ml, and the AUC of AFP was 0.879 (95% CI 0.850–0.907, sensitivity 67.8%, specificity of 91.23%; Fig 2A) at a cutoff value of 20 ng/ml; in cohort B, the AUC of DCP and AFP was0.914 (95% CI 0.885–0.943, sensitivity 80.5%, specificity 95.14%; Fig 2B, Table 1) and 0.814 (95% CI 0.777–0.852, sensitivity 62%, specificity of 87.85%), respectively. In both cohorts, a greater proportion of patients with HCC was positive for DCP than for AFP (Fig 2C and 2D).

**Table 1:** Results ofmeasurement of DCP, AFP, or both,^a^ in the diagnosis of HCC^b^.

	Cohort A						Cohort B					
	AUC(95%CI)	Sen (%)	Spe (%)	Accuracy	PPV (%)	NPV (%)	AUC(95%CI)	Sen (%)	Spe (%)	Accuracy	PPV (%)	NPV (%)
**HCC vs. DC+HC**												
**DCP**	**0.886 (0.855–0.917)**	**82.63**	**89.12**	**86.18%**	**86.28**	**86.10**	**0.914 (0.885–0.943)**	**80.50**	**95.14**	**88.59%**	**93.06**	**85.77**
**AFP**	**0.879 (0.850–0.907)**	**67.80**	**91.23**	**80.61%**	**86.49**	**77.38**	**0.814 (0.777–0.852)**	**62.00**	**87.85**	**76.29%**	**80.52**	**74.06**
**DCP+AFP**	**0.946 (0.928–0.964)**	**91.10**	**87.02**	**88.87%**	**85.32**	**92.19**	**0.924 (0.897–0.951)**	**84.50**	**93.93**	**89.71%**	**91.85**	**88.21**
**HCC vs. DC**												
**DCP**	**0.873 (0.841–0.905)**	**82.63**	**82.22**	**82.48%**	**89.04**	**73.03**	**0.913 (0.884–0.941)**	**82.63**	**90.72**	**84.98%**	**95.59**	**68.22**
**AFP**	**0.827 (0.792–0.863)**	**67.80**	**81.48**	**72.78%**	**86.49**	**59.14**	**0.691 (0.638–0.743)**	**62.00**	**69.07**	**64.31%**	**80.52**	**46.85**
**DCP+AFP**	**0.876 (0.843–0.910)**	**74.58**	**81.48**	**77.09%**	**87.56**	**64.71**	**0.840 (0.796–0.885)**	**78.50**	**93.81**	**83.05%**	**96.32**	**67.91**
**HCC size≤3cm vs DC+HC**												
**DCP**	**0.831 (0.770–0.893)**	**69.35**	**89.12**	**85.59%**	**58.11**	**93.04**	**0.843 (0.770–0.917)**	**58.70**	**95.14**	**89.42%**	**69.23**	**92.52**
**AFP**	**0.874 (0.824–0.924)**	**59.68**	**91.23**	**85.59%**	**59.68**	**91.23**	**0.781 (0.700–0.862)**	**56.52**	**87.85**	**82.94%**	**46.43**	**91.56**
**DCP+AFP**	**0.920 (0.890–0.950)**	**88.71**	**81.75**	**82.99%**	**51.40**	**97.08**	**0.861 (0.795–0.927)**	**73.91**	**90.69**	**88.05%**	**59.65**	**94.92**
**HCC size≤3cm vs DC**												
**DCP**	**0.815 (0.758–0.872)**	**69.35**	**82.2**	**78.17%**	**64.18**	**85.38**	**0.837 (0.771–0.903)**	**58.70**	**90.72**	**80.42%**	**75.00**	**82.24**
**AFP**	**0.811 (0.752–0.869)**	**59.68**	**81.48**	**74.62%**	**59.68**	**81.48**	**0.650 (0.555–0.745)**	**56.52**	**69.07**	**65.03%**	**46.43**	**91.56**
**DCP+AFP**	**0.861 (0.816–0.906)**	**90.32**	**66.67**	**74.11%**	**55.45**	**93.75**	**0.683 (0.567–0.799)**	**73.91**	**90.69**	**81.82%**	**59.65**	**94.92**
**HCC with cirrhosis vs. LC**												
**DCP**	**0.837 (0.789–0.885)**	**72.94**	**72.00**	**72.50%**	**74.70**	**70.13**	**0.932 (0.891–0.974)**	**90.16**	**90.24**	**90.20%**	**93.22**	**86.05**
**AFP**	**0.781 (0.726–0.836)**	**58.82**	**68.00**	**36.13%**	**67.57**	**59.30**	**0.800 (0.730–0.870)**	**54.10**	**80.49**	**64.71%**	**80.49**	**54.10**
**DCP+AFP**	**0.865 (0.822–0.909)**	**64.71**	**86.67**	**75.00%**	**84.62**	**68.42**	**0.939 (0.899–0.979)**	**88.52**	**92.50**	**90.10%**	**94.74**	**84.10**
**HCC without cirrhosis vs. LC**												
**DCP**	**0.871 (0.828–0.915)**	**84.48**	**72.00**	**79.58%**	**82.35**	**75.00**	**0.941 (0.906–0.975)**	**78.89**	**90.24**	**82.44%**	**94.67**	**66.07**
**AFP**	**0.711 (0.647–0.774)**	**64.66**	**68.00**	**65.97%**	**75.76**	**55.43**	**0.681 (0.601–0.761)**	**57.79**	**80.49**	**64.89%**	**86.67**	**46.48**
**DCP+AFP**	**0.875 (0.831–0.919)**	**83.62**	**82.67**	**83.25%**	**88.18**	**76.54**	**0.957 (0.926–0.988)**	**95.56**	**78.05**	**90.08%**	**90.52**	**88.89**

^*a*^The diagnostic cutoff values of serum DCP and AFP were 40mAU/ml and 20 ng/ml, respectively.

^*b*^HCC, hepatocellular carcinoma; DC, disease control; HC, healthy control; DCP, des-gamma-carboxy prothrombin; AFP, α-fetoprotein; AUC, area under curve; Sen, sensitivity; Spe, specificity; PPV, positive predictive value; NPV, negative predictive value; LC, liver cirrhosis.

More interestingly, 63.2%-76.3% of AFP-negative HCC had a positive DCP (Fig 2C and 2D). DCP showed higher accuracy than AFP by kappa reliability testing (kappa value: 0.72 vs. 0.60 in cohort A and 0.77 vs. 0.51 in cohort B).

In the assessment of high-risk population surveillance (Cohort B), DCP had a greater AUC, sensitivity and specificity than did AFP (Fig 2F, Table 1). The proportion of patients with a positive AFP in HL, LC and CHB was substantially higher than the proportion of patients with a positive DCP, and more DCP-negative cases were detected in AFP-positive, non-HCC patients (Fig 2G and 2H).

To assess whether the combined use of DCP and AFP was better than use of either of the two markers alone, mathematical models predicting the probability of HCC were created on the basis of an equation obtained by binary logistic regression in both cohorts. The regression equations for all comparisons are shown in S2 Table. The diagnostic accuracy improved 1.12–2.69% (vs. DCP) and 8.26–13.42% (vs. AFP) when the two tests were combined (Fig 2, Table 1).

### The diagnostic performance of DCP in identifying early HCC

**Fig 3:**
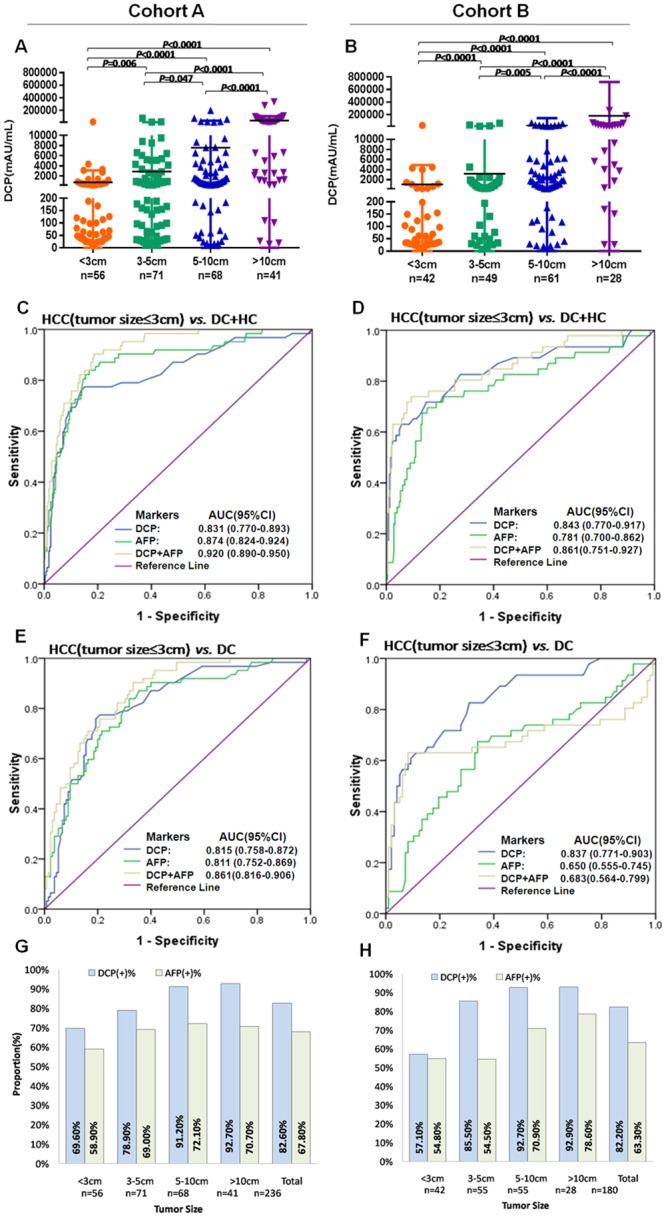
Diagnostic capability of DCP for detecting early HCC with a solitary tumor smaller than 3cm. (A and B) The concentrations of serum DCP in HCC according to tumor size (<3cm, ≥3 and ≤5cm, >5 and ≤10cm, or >10cm). (C and D) ROC curves of DCP, AFP, or their combination in HCC with a solitary small tumor (≤3cm) versus all controls (HCs and DC). (E and F) ROC curves of DCP, AFP, or their combination in HCC with a solitary small tumor (≤3cm) versus disease controls (G and H). The positive rates of serum DCP and AFP in HCC according to tumor size (<3cm, ≥3 and ≤5cm, >5 and ≤10cm, or >10cm). DCP, des-gamma-carboxy prothrombin; AFP, alpha-fetoprotein; HCC, hepatocellular carcinoma; HC, healthy control; DC, disease control; ROC, receiver operating characteristics; AUC, area under the curve; 95% CI, 95% confidence interval.

A solitary tumor less than 3 cm was usually defined clinically as early-stage HCC [8]. As shown in Fig 3, the level of DCP was positively correlated with tumor size (Fig 3A and 3B). The AUC of DCP in differentiating early HCC from DCs was 0.831 in cohort A (95% CI 0.77–0.893), with a sensitivity of 69.35% and a specificity of 89.12%, and 0.837 in cohort B (95% CI 0.771–0.903), with a sensitivity of 58.7% and a specificity of 90.72%. DCP had a better AUC than AFP (AFP: 0.811, 95% CI 0.752–0.869 in cohort A; 0.650, 95% CI 0.555–0.745 in cohort B, *P*<0.0001, Fig 3E and 3F, Table 1).

Additionally, the positive rates of DCP were higher than those of AFP in HCC with different tumor sizes, and in cohort A, the total positive rate of DCP in HCC was 82.6%, which was remarkably higher than that of AFP (67.8%). This diagnostic superiority was also found in cohort B (Fig 3G and 3H).

### The performance of DCP in discriminating HCC from liver cirrhosis

**Fig 4:**
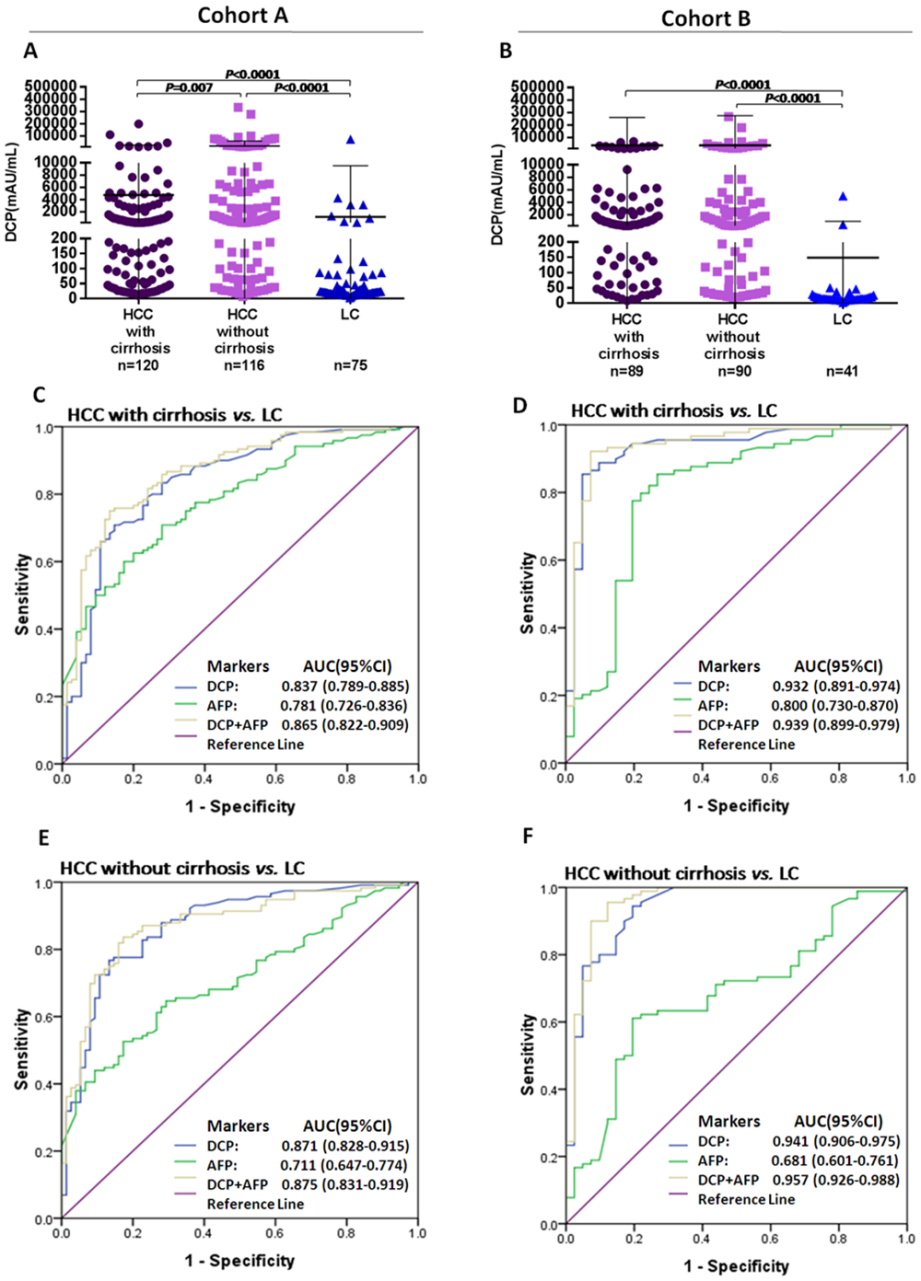
Diagnostic capability of DCP in discriminating HCC from LC. (A and B) The concentration of DCP in patients with LC and patients with HCC with or without a cirrhosis background. (C and D) ROC curves of DCP, AFP, or their combination in HCC patients with a cirrhosis background versus LC patients. (E and F) ROC curves of DCP, AFP, or their combination in HCC patients without a cirrhosis background versus LC patients. DCP, des-gamma-carboxy prothrombin; AFP, alpha-fetoprotein; LC, liver cirrhosis; HCC, hepatocellular carcinoma patients; ROC, receiver operating characteristics; AUC, area under the curve; 95% CI, 95% confidence interval.

In cohort A, in differentiating HCC from LC, the AUC of DCP (0.837, 95% CI 0.789–0.885) was better than that of AFP (0.781, 95% CI 0.726–0.836), and the combination of these two markers increased the AUC further to 0.865 (95% CI 0.822–0.909) (Fig 4C, Table 1).

In discriminating HCC without cirrhosis from LC, the AUC of DCP (0.871, 95% CI 0.828–0.915) was much better than that of AFP (0.711, 95% CI 0.647–0.774), and the combination of the two markers had a similar AUC (0.875, 95% CI 0.831–0.919) (Fig 4E, Table 1). Although in cohort A the DCP in HCC without cirrhosis was higher than that in HCC with cirrhosis (median [range], 939 [7–333568] mAU/ml vs290 [11–198267] mAU/ml, *P* = 0.007) (Fig 4A), this significant difference was not found in cohort B (Fig 4B). Analysis of cohort B substantiated these results, with an even better diagnostic performance for DCP (Fig 4D and 4F, Table 1).

### The diagnostic performance of DCP in AFP-negative HCC

Although the positive rate of DCP in AFP-positive HCC was higher than that in AFP-negative HCC [A: 85.6% vs. 76·3%; B: 91.1% vs. 63.2%, Fig 2C and 2D, S2A and S2B Fig], there was no correlation between AFP and DCP because the correlation indices (R^2^ values) were only 0.061 in cohort A and 0.163 in cohort B (S2C and S2D Fig).

**Fig 5:**
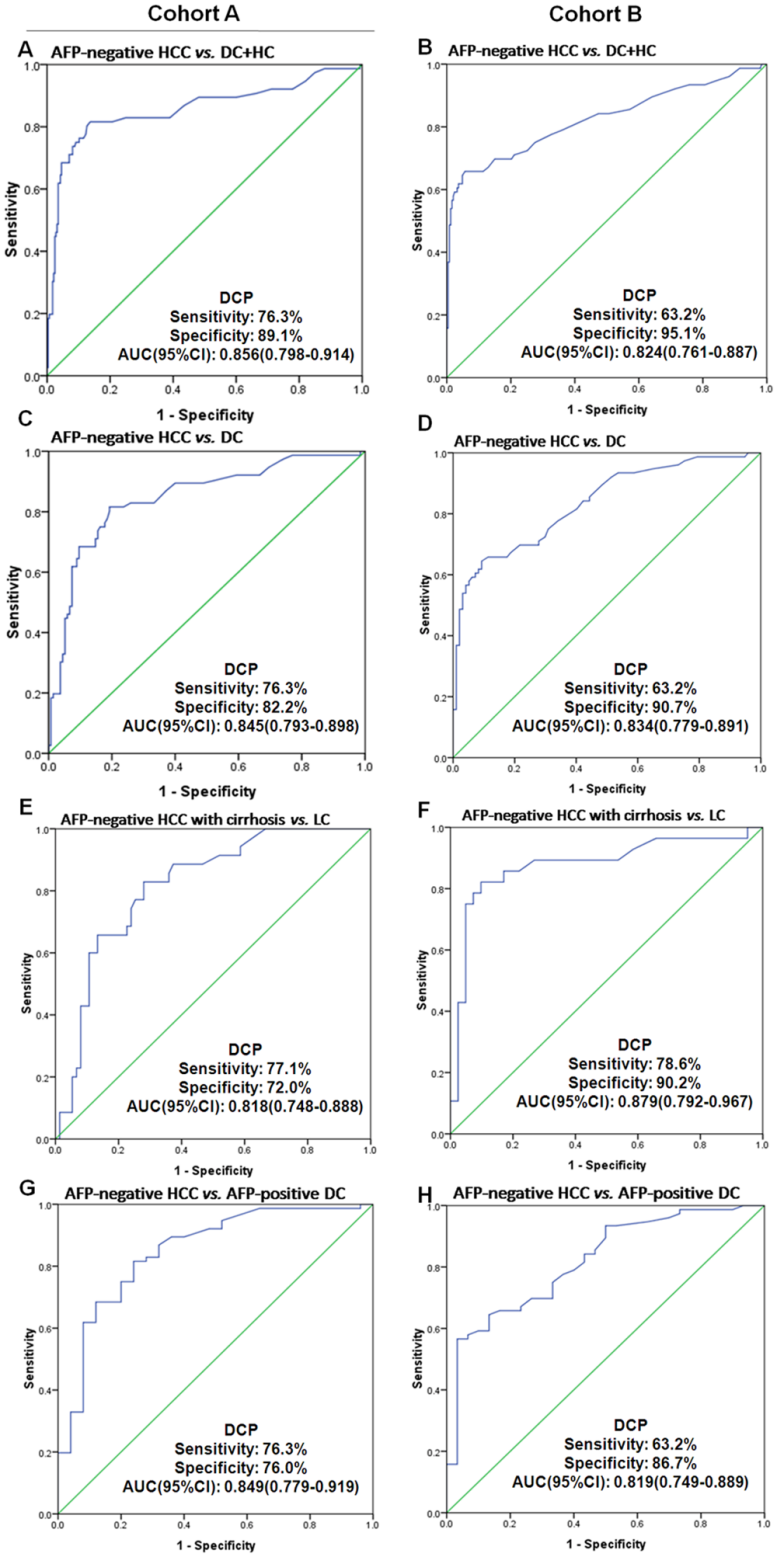
Diagnostic capability of DCP in AFP-negative HCC. (A and B) ROC curve of DCP in AFP-negative HCC versus all control subjects (HCs and DC). (C and D) ROC curve of DCP in AFP-negative HCC patients versus control subjects (DC). (E and F) ROC curve of DCP in AFP-negative HCC patients with a cirrhosis background versus LC patients. (G and H) ROC curve of DCP in AFP-negative HCC versus AFP-positive control subjects (DC). DCP, des-gamma-carboxy prothrombin; AFP, alpha-fetoprotein; HCC, hepatocellular carcinoma; DC, disease control; HC, healthy control; AFP-negative, serum AFP≤20 ng/ml; AFP-positive, serum AFP>20 ng/ml; ROC, receiver operating characteristics; AUC, area under the curve; 95% CI, 95% confidence interval.

In differentiating AFP-negative HCC from all control subjects, the AUC of DCP was 0.856 (95% CI 0.798–0.914, sensitivity: 76.3%, specificity: 89.1%, Fig 5A). This performance was confirmed in cohort B (Fig 5B). In discriminating AFP-negative HCC from benign liver diseases and liver metastasis (HL, LC and MT), a similar AUC (0.845, 95% CI 0.793–0.898, sensitivity: 76.3%, specificity: 82.2%, Fig 5C) was found. In differentiating AFP-negative HCC from the HCC risk population (CHB and LC) in cohort B, once again, a similar AUC (0.834, 95% CI 0.779–0.891, sensitivity: 63.2%, specificity: 90.7%, Fig 5D) was revealed.

Furthermore, the diagnostic capability of DCP in discriminating AFP-negative HCC patients with a cirrhosis background from LC patients was investigated. The ROC curve is shown in Fig 5E and indicates that DCP could distinguish AFP-negative HCC patients with cirrhosis from LC patients at a sensitivity of 77.1% and specificity of 72.0%. The diagnostic efficacy was also found in cohort B (Fig 5F).

In addition, the diagnostic performance of DCP in distinguishing AFP-negative HCC from AFP-positive benign liver disease was assessed. In cohort A, the AUC of DCP was 0.849 (95% CI 0.779–0.919, sensitivity 76.3%, specificity 76.0%; Fig 5G). The performance was proven in cohort B (Fig 5H).

### The relationship between DCP and clinical features of HCC

The enrolled HCC patients were divided into high and low DCP concentration groups according to their median DCP value (S3 Table). Among the several clinical features in the two cohorts (A and B), tumor size and AST were significantly different between the high and low DCP groups according to Pearson’s χ^2^ test (*P*<0.01, S3 Table). In cohort A, a higher DCP was significantly associated with tumor encapsulation (*P*<0.001), satellite lesion (*P* = 0.044) and vascular tumor thrombus (*P* = 0.018). In cohort B, the above observations confirmed that higher DCP was significantly associated with tumor number (*P* = 0.048), tumor differentiation (*P* = 0.005) and TNM stage (*P* = 0.006).

### The prognostic value of DCP for HCC after treatment

**Fig 6:**
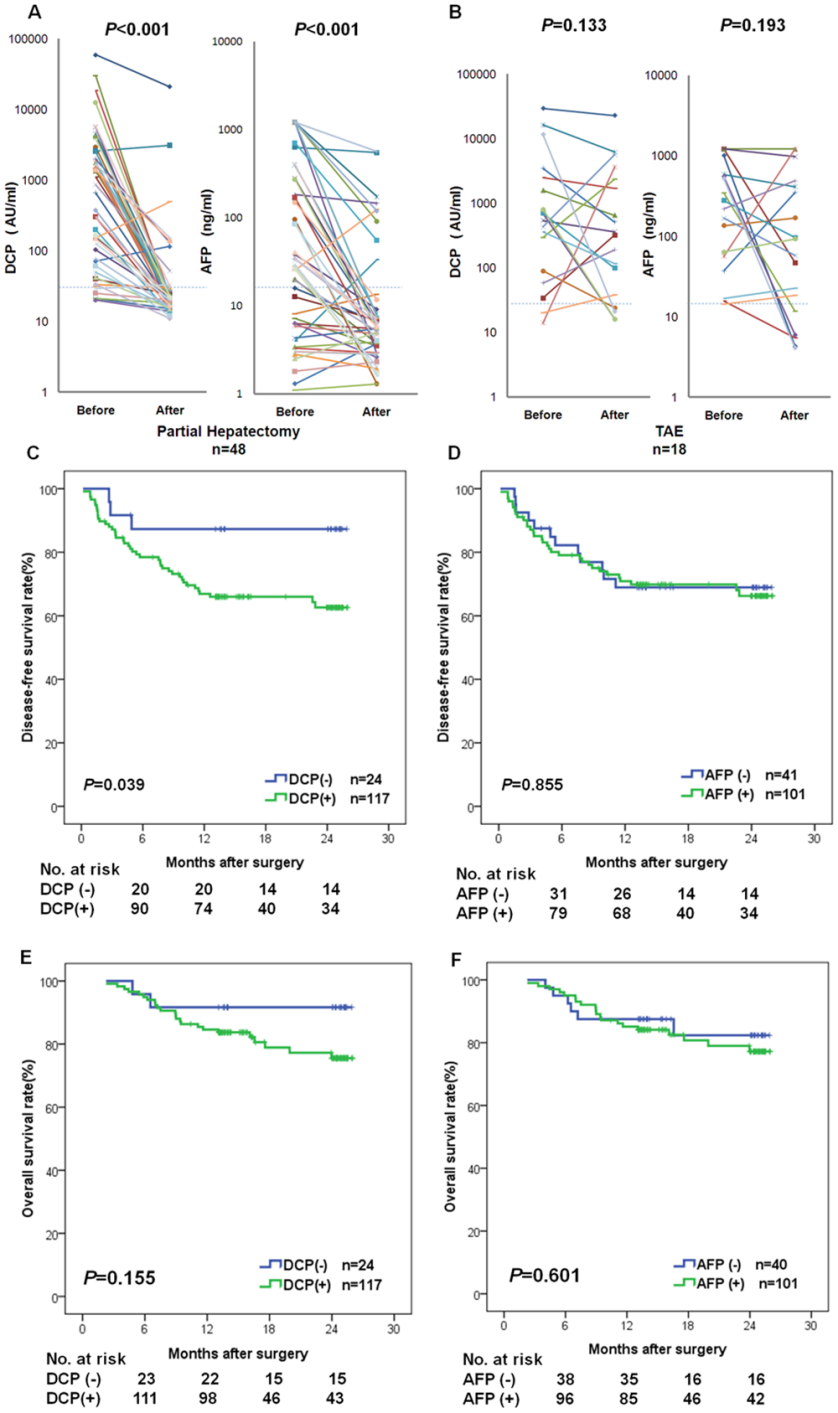
Longitudinal evaluation of DCP and AFP in HCC. (A) Serum DCP and AFP levels in HCC before and after radical partial hepatectomy therapy. (B) Serum DCP and AFP levels in HCC before and after TAE therapy. (C and D) Disease-free survival (DFS) rates were compared between the positive and negative DCP/AFP groups by Kaplan-Meier analysis. (E and F) Overall survival (OS) rates were compared between the positive and negative DCP/AFP groups by Kaplan-Meier analysis. DCP, des-gamma-carboxy prothrombin; AFP, alpha-fetoprotein; TAE, transcatheter arterial embolization; DCP (-), patients with negative DCP (serum DCP≤40 mAU/ml); DCP (+), patients with positive DCP (serum DCP >40 mAU /ml); AFP(-), patients with negative AFP (serum AFP≤20 ng/ml); AFP (+), patients with positive AFP (serum AFP>20 ng/ml).

The tumor marker values (DCP and AFP) of patients with HCC before surgery and 1 or 2 months (median 40 days) after treatment in whom no recurrence was detected until 6 months post-surgery are depicted in Fig 6A and 6B. The median concentration of DCP and AFP before curative hepatectomy was 515.5 mAU/ml (range 20–58878) and 31.8 ng/ml (range 1.1–1210), and the DCP values [20.5 mAU/ml (range 11–20834), *P*<0.001] dropped significantly after surgery, with a steeper slope compared to that of AFP (5.5 ng/ml [range 1.3–565.7], *P*<0.001, Fig 6A). DCP and AFP were not changed significantly in patients with non-curative treatment (TAE therapy, *P*>0.05, Fig 6B).

A total of 141 patients in cohort A were followed up for 26 months (median, 15.8 months) after hepatectomy from June 2011 to July 2014. The primary endpoint (death) was observed in 18% (26/141). This group of 141 patients was split into two subgroups according to the positivity/negativity of DCP [DCP (-), n = 24; DCP (+), n = 117]. There were significant differences in AFP concentration, tumor size, and TNM stage between DCP-negative and DCP-positive subjects (*P*<0.05, S4 Table). The median disease-free survival (DFS) in the DCP (+) group was significantly shorter than that in the DCP (-) group [DCP (+) group, 13.8 months (0.2 to 25.9 months); DCP (-) group, 24.1 months (2.7 to 25.9 months), *P* = 0.036, Fig 6C]. However, there was no significant difference in median overall survival (OS) between the DCP (+) and DCP (-) groups [DCP (+) group, 15.6 months (2.3 to 25.9 months); DCP (-) group, 24.2 months (4.8 to 25.9 months), *P* = 0.155, Fig 6E]. In comparison, no significant difference was observed between the AFP (+) and AFP (-) groups in either DFS or OS (*P*>0.05, Fig 6D and 6F). Tumor size and TNM stage were the two independent risk factors for DFS and OS by multivariate analysis (S5 and S6 Tables).

### Assay reproducibility

To evaluate the stability of the DCP assay, three levels (high, median, low) of DCP were determined ten times in a single run to evaluate intra-assay variation. Inter-assay variation was measured by testing three levels (high, median, low) of DCP in two separate consecutive runs during ten days (a total of 20 tests per DCP level). The mean, standard deviation (SD) and coefficient of variation (CV) for both intra-assay and inter-assay variations were calculated separately for each DCP level. The coefficient of variation (CV) values for intra- and inter-assay variability of DCP were low, ranging from 2.44% to 0.55% and 2.59% to 1.32%, respectively (S7 Table).

## Discussion

In the present study, we constructed a multicenter study from four large academic medical centers from three different regions of China. The results demonstrated that the accuracy and sensitivity of DCP were higher than that of AFP at the cutoff levels of 40 mAU/ml (DCP) and 20 ng/ml (AFP). The higher accuracy and sensitivity (Table 1), the increased kappa consistency (kappa value: 0.72 vs. 0.60 in cohort A and 0.77 vs. 0.51 in cohort B), and the elevated positive proportion of DCP are similar and even better than most findings in HCV-related HCCs [27,28], indicating that DCP is suitable and superior to AFP for HBV-related HCC surveillance.

In addition to the general improved diagnostic performance of DCP, we further found that compared to AFP, DCP had a superior performance in: 1) the identification of HCC from HBV-related non HCC [DCP: AUC 0.837 (95% CI: 0.771–0.903) vs. AFP 0.650 (95%CI: 0.555–0.745)], 2) the differential diagnosis between HCC and liver cirrhosis, regardless of the presence or absence of a cirrhotic background (Fig 4), and 3) the high capacity for identifying HCC with negative AFP (Fig 5).

Early identification of HCC is important and will surely increase the likelihood of successful treatment and improve prognosis [4]. The enhanced differential diagnostic efficacy of DCP to distinguish between HCC and liver cirrhosis and its high capacity for identifying HCC with negative AFP are important complements to AFP because an elevation in AFP could occur in approximately 11–47% of subjects with liver cirrhosis, and a false-negative AFP appeared in 30–40% of subjects with HCC [29]. Additionally, DCP could provide an effective marker for recurrence monitoring after curative surgery if the AFP is negative before treatment [3]. Some of our above findings differed from the findings of previous research studies [28,30,31]. Three major reasons might contribute to the differences: 1) etiological difference of the enrolled subjects (e.g., 51.5% HCV-related HCC by Marrero [28] vs. 90% HBV-related in this study), 2) disease proportion differences in enrolled cohorts, and 3) different methods of DCP detection used in the different studies [e.g., ELISA (Eisai Co, Tokyo, Japan [28]) vs. CLEIA (Fujirebio, Tokyo, Japan)].

Furthermore, we validated that higher DCP levels were significantly associated with aggressive tumor behavior, including larger tumor size, increased tumor number, increased vascular invasion, later tumor stage and poor liver function (S3 Table, Fig 3). It was reported that DCP may induce the proliferation of malignant hepatocytes through binding with Met and the subsequent stimulation of Met-Janus kinase-signal transducers and activators of the transcription 3 pathway as the signaling pathway [32]. DCP was regarded as a novel vascular endothelial growth factor with potent mitogenic and migration activities in both autocrine and paracrine manners because it was considered to stimulate proliferation and migration of vascular endothelial cells through the EGFR-MAPK pathway [12,33,34]. These molecular functions of DCP support our finding that serum DCP was positively correlated with aggressive tumor characteristics.

In addition to the above cross-sectional observations, we also conducted longitudinal testing. There was a striking decrease in DCP concentration after curative surgery (Fig 6A), but no similar decrease was found in TAE, a palliative therapy. The temporal decrease in DCP 1–7 days after curative surgery was more evident (steeper slope as was shown in S3 Fig) than the decrease in AFP because the half-life of DCP is shorter than that of AFP. Furthermore, in the follow-up study, positive DCP subjects had a much worse DFS, whereas in the same follow-up cohort, AFP was not correlated with DFS and OS. The above longitudinal studies validate that DCP is superior to AFP for dynamic monitoring of HCC in treatment response and correlates well with recurrence after curative therapy. However, in multivariate analysis, only tumor size and advanced TNM stage were independent predictors of DFS and OS; both DCP and AFP failed to be independent predictors of DFS and OS. In future studies, a larger sample size and longer follow-up period are required to elucidate the predictive value of DCP.

This study is noteworthy for several reasons. This was a carefully designed, large-scale, multi-centre study that evaluated the clinical diagnostic value of DCP for HBV-related HCC surveillance. Two independent cohorts (A and B) were enrolled from three different regions of China. Particularly, both case-control and longitudinal temporal designs were conducted. There has been only one previous study in China that has addressed the value of DCP at a single time point involving 336 HCC patients and 252 liver diseases controls, and the main conclusion was that the combination of DCP and AFP had a total sensitivity of 84%, which was higher than that of either DCP (74%) or AFP (62%) alone [23]. In our study, there was a similar disease constitution in cohort B (200 HCC patients and 97 liver diseases control),and the results showed that the combination of DCP and AFP had a total specificity of 93.81%, which was higher than that of either DCP (90.72%) or AFP (69.07%) alone, whereas the sensitivity of DCP (82.63%) alone was higher than that of either the combination of DCP and AFP (78.5%) or AFP (62%) alone (Table 1). A second advantage of this study was that control patients with a broad spectrum of liver disease controls were enrolled to make differential diagnoses of benign-malignant liver conditions and primary-metastatic cancers and to perform HCC surveillance in patients with high-risk HBV-related liver diseases. The disease controls included liver metastasis (MT), liver cirrhosis (LC) and hemangiomas of liver (HL) in cohort A to evaluate differential diagnoses and chronic hepatitis B (CHB) and liver cirrhosis (LC) in cohort B to assess HCC surveillance in the HBV infection (high-risk) population. The performance of DCP was carefully evaluated in identifying AFP-negative HCC and in differentiating AFP-positive non-HCC. A recent study reported that in chronic hepatitis patients with AFP<20 ng/ml, DCP showed a high specificity of 100% in patients who combined with HCC, using a microchip capillary electrophoresis and liquid-phase binding assay [24]. In our research, DCP also showed a high specificity of 90.7% in distinguishing patients with HCC with AFP<20 ng/ml from patients with CHB and LC. The two above studies consistently indicated that DCP was highly important in determining AFP-negative HCC. Moreover, the dynamic monitoring of DCP after treatment and the correlation with recurrence after curative surgery provided in this study are part of the important clinical information needed by clinicians to assess the treatment response and help clinicians manage disease, predict the disease process and establish a personalized follow-up strategy.

However, there are still some limitations to this study. Both the number and the duration of follow-up cases are insufficient. Due to the time limitation (till drafting in July 2014), the longest follow-up duration available was less than 3 years, which might not be long enough to assess the predictive value of DCP and the independent risk factors affecting DFS and OS. Larger sample sizes and longer follow-up periods are required in future studies to further elucidate its predictive value. Furthermore, obstructive jaundice, vitamin K deficiency, alcohol intake, or taking warfarin might induce aberrant increases in serum DCP [27]. Such interference on DCP was not evaluated in this study; an improved DCP assay might overcome some of these defects [35]. Finally, due to clinical infeasibility, the dynamic changes in DCP from CHB/LC to HCC need to be assessed in future studies.

In conclusion, we demonstrated that serum DCP can be used for the differential diagnosis and surveillance of high-risk subjects with HBV-related HCC. DCP is especially promising in compensating for the insufficiency of AFP in identifying cases of AFP-negative HCC and in excluding cases of AFP-positive non-HCC. Thus, DCP is a complement to and might be superior to AFP in HCC surveillance, early diagnosis, treatment response and recurrence monitoring. The combination of DCP and AFP will provide more solid evidence for HCC surveillance, treatment and follow-up monitoring.

## Supporting Information

S1 Fig DCP and AFP concentrations in cohorts A and B. (A) DCP in cohort A. (B) DCP in cohort B. (C) AFP in cohort A. (D) AFP in cohort B. Black horizontal lines are means, and error bars are SEs. DCP, des-gamma-carboxy prothrombin; AFP, α-fetoprotein; HCC, hepatocellular carcinoma; MT, liver metastasis; LC, liver cirrhosis; HL, hemangiomas of the liver; CHB, chronic hepatitis B virus infection; HC, healthy control.
(TIF)

S2 Fig Correlation between DCP and AFP in HCC. (A and B) The concentration of DCP in HCC with negative and positive AFP. (C and D) A scatter diagram of the correlation between DCP and AFP in HCC. DCP, des-gamma-carboxy prothrombin; AFP, alpha-fetoprotein; HCC, hepatocellular carcinoma; DC, disease control; HC, healthy control; AFP-, patients with negative AFP (serum AFP≤20 ng/ml); AFP+, patients with positive AFP (serum AFP>20 ng/ml); R^2^, related index.
(TIF)

S3 Fig The temporal change of DCP and AFP in 6 HCC patients after curative surgery on days 1, 3 and 7. (A) The level of DCP ondays1, 3 and 7 after curative hepatectomy. (B) The level of AFP on days 1, 3 and 7 after curative hepatectomy.
(TIF)

S1 Table The clinicopathologic features of subjects in cohorts A and B. (DOC)

S2 Table The regression equations and optimum probabilities of the combination of DCP and AFP. (DOC)

S3 Table Correlation between DCP and clinicopathologic characteristics of HCC patients in cohorts A and B. (DOC)

S4 Table Differences in clinical features between the DCP-negative and -positive groups. (DOC)

S5 Table Univariate and multivariate analysis of risk factors affecting disease-free survival. (DOC)

S6 Table Univariate and multivariate analysis of risk factors affecting overall survival. (DOC)

S7 Table Assay reproducibility. (DOC)
