# Supplementary figures and images for: Diagnostic Evaluation of Des-Gamma-Carboxy Prothrombin versus α-Fetoprotein for Hepatitis B Virus-Related Hepatocellular Carcinoma in China: A Large-Scale, Multicentre Study
Source: PLoS One. 2016 Apr 12;11(4):e0153227. doi: 10.1371/journal.pone.0153227 (PMC4829182; doi:10.1371/journal.pone.0153227)

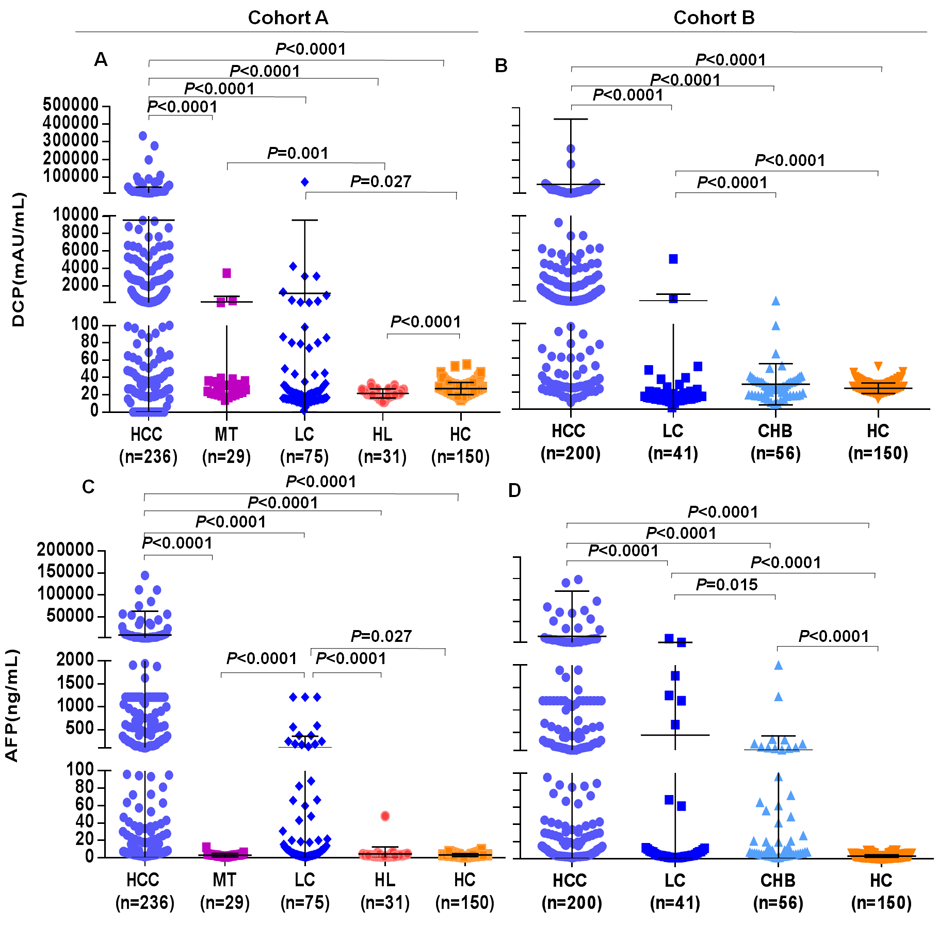

Supplement: S1 Fig — (A) DCP in cohort A. (B) DCP in cohort B. (C) AFP in cohort A. (D) AFP in cohort B. Black horizontal lines are means, and error bars are SEs. DCP, des-gamma-carboxy prothrombin; AFP, α-fetoprotein; HCC, hepatocellular carcinoma; MT, liver metastasis; LC, liver cirrhosis; HL, hemangiomas of the liver; CHB, chronic hepatitis B virus infection; HC, healthy control. (TIF) [file pone.0153227.s001.tif]

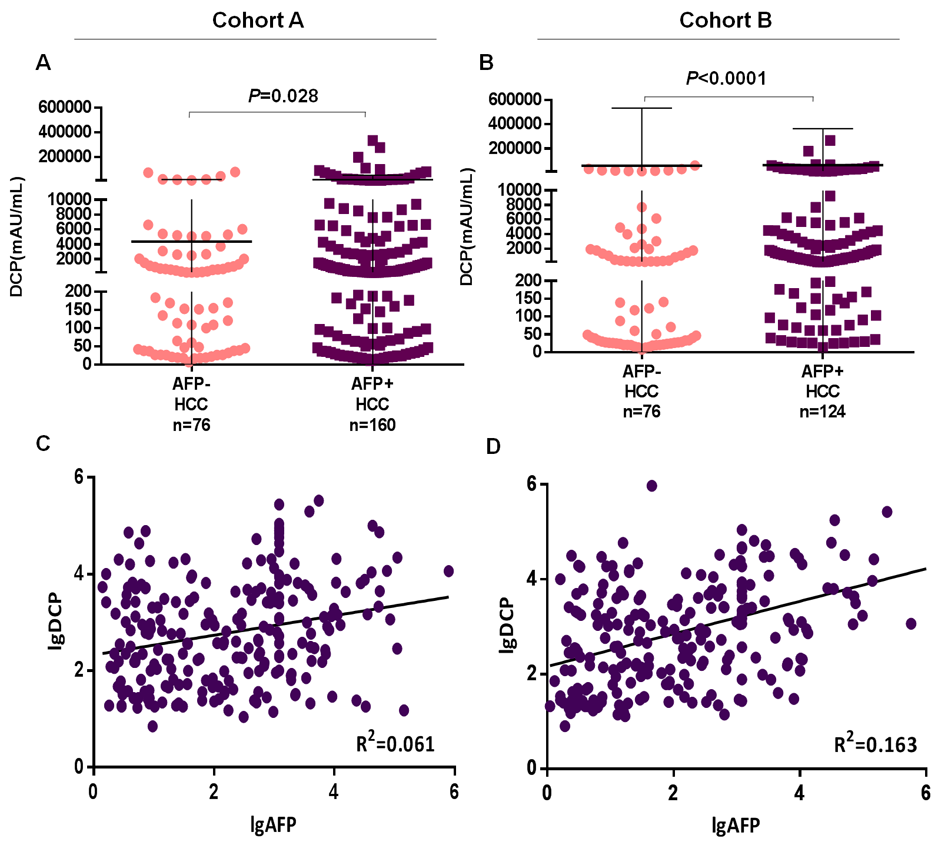

Supplement: S2 Fig — (A and B) The concentration of DCP in HCC with negative and positive AFP. (C and D) A scatter diagram of the correlation between DCP and AFP in HCC. DCP, des-gamma-carboxy prothrombin; AFP, alpha-fetoprotein; HCC, hepatocellular carcinoma; DC, disease control; HC, healthy control; AFP-, patients with negative AFP (serum AFP≤20 ng/ml); AFP+, patients with positive AFP (serum AFP>20 ng/ml); R2, related index. (TIF) [file pone.0153227.s002.tif]

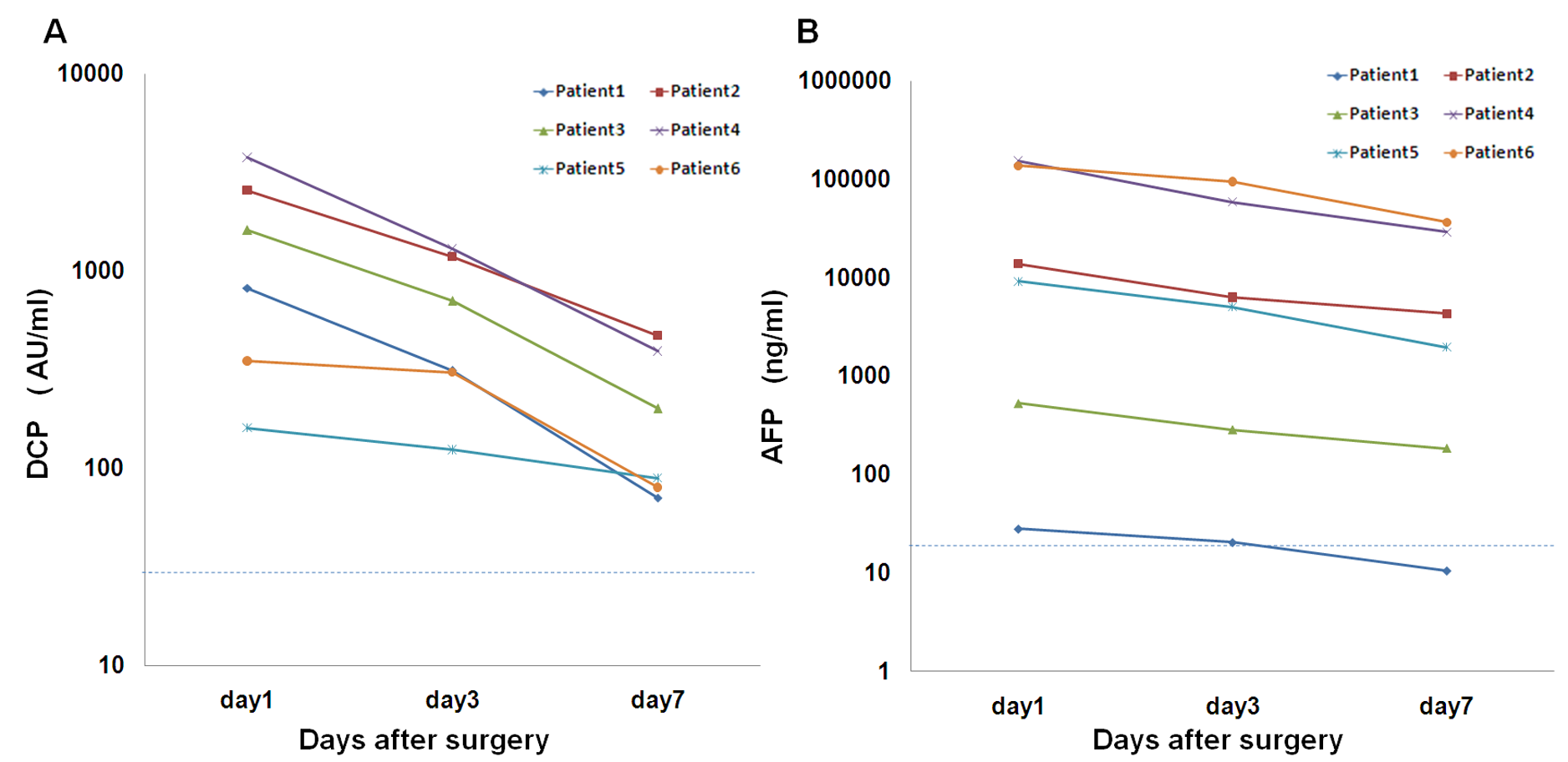

Supplement: S3 Fig — (A) The level of DCP ondays1, 3 and 7 after curative hepatectomy. (B) The level of AFP on days 1, 3 and 7 after curative hepatectomy. (TIF) [file pone.0153227.s003.tif]
