# Supplementary material for: Diagnostic Evaluation of Des-Gamma-Carboxy Prothrombin versus α-Fetoprotein for Hepatitis B Virus-Related Hepatocellular Carcinoma in China: A Large-Scale, Multicentre Study
Source: PLoS One. 2016 Apr 12;11(4):e0153227. doi: 10.1371/journal.pone.0153227 (PMC4829182; doi:10.1371/journal.pone.0153227)
Supplement: S1 Table — (DOC) [file pone.0153227.s004.doc]

**S1 Table. The clinicopathologic features of subjects in cohort A and B**

| **Features** | **Test (n=604)** | | | | |  |  | **Validation(n=447)** | | | |  |
| --- | --- | --- | --- | --- | --- | --- | --- | --- | --- | --- | --- | --- |
|  | **HC** | **HL** | **LC** | **MT** | **HCC** | ***P* value** |  | **HC** | **CHB** | **LC** | **HCC** | ***P* value** |
|  | **n=150** | **n=31** | **n=75** | **n=29** | **n=236** |  |  | **n=150** | **n=56** | **n=41** | **200** |  |
| **Age, mean (range), y** | **48.4 (21, 76)** | **45 (26, 59)** | **53.3 (32, 77)** | **54.2 (38, 77)** | **51 (22, 74)** | ***P*<0.0001** |  | **48.5 (24, 80)** | **40.6 (18, 76)** | **53.1 (24, 84)** | **50.3 (10, 82)** | ***P*<0.0001** |
| **Male-to-female ratio** | **5 (125/25)** | **0.55 (11/20)** | **1.78 (48/27)** | **3.14 (22/7)** | **5.2 (198/38)** | ***P*<0.0001** |  | **1 (75/75)** | **3.3 (43/13)** | **2.7 (30/11)** | **5.8 (171/29)** | ***P*<0.0001** |
| **Positive for HBsAg** | **0% (0/150)** | **9.7% (3/31)** | **60% (45/75)** | **3.4% (1/29)** | **90.7% (214/236)** | ***P*<0.0001** |  | **0% (0/150)** | **100% (56/56)** | **78% (32/41)** | **89% (178/200)** | ***P*<0.0001** |
| **Laboratory parameters [mean ± SD ,or median (range)]** | | | | | | | | | | | | |
| **Total protein (g/L)** | **76.0±3.8** | **71±6.7** | **62.0±17.6** | **69.0±6.1** | **67.0±6.2** | ***P*<0.0001** |  | **74.5±3.8** | **67.0±8.4** | **63.1±8.8** | **67.0±6.9** | ***P*<0.0001** |
| **Albumin (g/L)** | **46.6±2.3** | **43.7±4.3** | **33.7±6.4** | **41.2±4.0** | **41.5±4.0** | ***P*<0.0001** |  | **46.2±2.5** | **37.5±7.1** | **31.7±6.1** | **40.6±4.7** | ***P*<0.0001** |
| **ALT (IU/L)** | **21.2±11.3** | **12 (5, 867)** | **38 (10, 464.7)** | **30 (10, 328.3)** | **36 (8, 1253)** | ***P*<0.0001** |  | **16.7±7.4** | **81 (14, 3324)** | **40.7 (11, 385)** | **42.9±35.4** | ***P*<0.0001** |
| **AST (IU/L)** | **21.6±5.3** | **16 (9, 824)** | **48 (14, 590)** | **25.8 (13, 205.6)** | **35 (13, 1078)** | ***P*<0.0001** |  | **19.8±4.5** | **86 (17, 3541)** | **51.4 (21, 422)** | **47.5±37.7** | ***P*<0.0001** |
| **AFP (ng/ml)** | **3.3±1.7** | **2.9 (1.5, 48)** | **8.4(0.8,580.7)** | **3.2±2.1** | **154.7 (1.4,800000)** | ***P*<0.0001** |  | **3.1±1.5** | **7.32 (1.3, 2000)** | **4.59 (1.24, 10810)** | **70.4 (1.1, 1329100)** | ***P*<0.0001** |
| **DCP (mAU/ml)** | **27.1±7.1** | **21.5±5.3** | **22 (2, 72377)** | **27 (14, 3475)** | **490 (7, 333568)** | ***P*<0.0001** |  | **24.6±6.2** | **25 (6, 169)** | **15 (2, 5001)** | **774 (8,4146590)** | ***P*<0.0001** |

**Abbreviations: HC, healthy controls; HL, hemangiomas of liver; LC, liver cirrhosis; MT, metastatic hepatic carcinoma; CHB, chronic hepatitis B; HCC, hepatocellular carcinoma;**

**HBsAg, hepatitis B surface antigen; ALT, alanine aminotransferase; AST, aspartate transaminase; AFP, α-fetoprotein; DCP, des-gamma-carboxy prothrombin.**
