# Supplementary material for: Diagnostic Evaluation of Des-Gamma-Carboxy Prothrombin versus α-Fetoprotein for Hepatitis B Virus-Related Hepatocellular Carcinoma in China: A Large-Scale, Multicentre Study
Source: PLoS One. 2016 Apr 12;11(4):e0153227. doi: 10.1371/journal.pone.0153227 (PMC4829182; doi:10.1371/journal.pone.0153227)
Supplement: S2 Table — (DOC) [file pone.0153227.s005.doc]

S2 Table. The regression equations and optimum probabilities of the combination of DCP and AFP

|  | **Cohort A** |  | **Cohort B** | |
| --- | --- | --- | --- | --- |
| **Group** | **Equation** | **Cutoff** | **Equation** | **Cutoff** |
| **HCC vs. DC+HC** | **2.148lgDCP+1.545lgAFP-6.016** | **-1.1706** | **3.636lgDCP+0.652lgAFP-7.211** | **-0.7588** |
| **HCC vs. DC** | **1.751lgDCP+1.128lgAFP-4.414** | **0.9389** | **3.179lgDCP+0.05lgAFP-5.02** | **0.5031** |
| **HCC (Size≤3cm） vs. DC+HC** | **1.384lgDCP+1.519lgAFP-5.546** | **-2.2423** | **3.055lgDCP+0.79lgAFP-7.266** | **-1.7022** |
| **HCC (Size≤3cm） vs. DC** | **1.099lgDCP+1.133lgAFP-4.157** | **-1.592** | **2.617lgDCP+0.42lgAFP-5.173** | **-0.5637** |
| **HCC with cirrhosis  vs. LC** | **1.367lgDCP+0.911lgAFP-3.705** | **0.4524** | **2.788lgDCP+0.42lgAFP-4.907** | **-0.0417** |
| **HCC without cirrhosis  vs. LC** | **1.554lgDCP+0.623lgAFP-3.791** | **-0.0241** | **3.451lgDCP-0.316lgAFP-4.794** | **-0.2967** |
| **The cutoff values were determinate from ROC curves by maximizing the sum of sensitivity and specificity.**  **Abbreviations: DCP, des-gamma-carboxy prothrombin; AFP, alpha-fetoprotein; HCC, hepatocellular carcinoma; DC, disease controls; HC, healthy controls** | | | | |
