# Supplementary material for: Diagnostic Evaluation of Des-Gamma-Carboxy Prothrombin versus α-Fetoprotein for Hepatitis B Virus-Related Hepatocellular Carcinoma in China: A Large-Scale, Multicentre Study
Source: PLoS One. 2016 Apr 12;11(4):e0153227. doi: 10.1371/journal.pone.0153227 (PMC4829182; doi:10.1371/journal.pone.0153227)
Supplement: S3 Table — (DOC) [file pone.0153227.s006.doc]

**S3 Table. Correlation between DCP and clinicopathologic characteristics of HCC*^a^* patients in A and B cohorts*^b^***

|  |  |  | **Cohort A (n=236)** | |  |  | **Cohort B (n=200)** |  |
| --- | --- | --- | --- | --- | --- | --- | --- | --- |
| **Variable** |  | **Low** | **High** | ***P* value** |  | **Low** | **High** | ***P* value** |
|  |  | **118** | **118** |  |  | **100** | **100** |  |
| **Age(y)** |  |  |  |  |  |  |  |  |
|  | **≤50** | **56** | **57** | **0.896** |  | **48** | **56** | **0.258** |
|  | **＞50** | **62** | **61** |  |  | **52** | **44** |  |
| **Gender** |  |  |  |  |  |  |  |  |
|  | **Male** | **97** | **101** | **0.479** |  | **83** | **88** | **0.876** |
|  | **Female** | **21** | **17** |  |  | **17** | **12** |  |
| **HBsAg** |  |  |  |  |  |  |  |  |
|  | **Positive** | **106** | **108** | **0.965** |  | **86** | **92** | **0.881** |
|  | **Negative** | **12** | **10** |  |  | **11** | **11** |  |
| **HBeAg** |  |  |  |  |  |  |  |  |
|  | **Positive** | **30** | **37** | **0.374** |  | **25** | **27** | **0.778** |
|  | **Negative** | **82** | **78** |  |  | **65** | **64** |  |
|  | **Missing** | **28** |  |  |  |  |  |  |
| **Cirrhosis** |  |  |  |  |  |  |  |  |
|  | **Yes** | **68** | **52** | **0.05** |  | **45** | **44** | **0.136** |
|  | **No** | **50** | **66** |  |  | **44** | **46** |  |
|  | **Missing** | **21** |  |  |  |  |  |  |
| **AFP(ng/ml)** | |  |  |  |  |  |  |  |
|  | **≤20** | **43** | **33** | **0.164** |  | **49** | **28** | **0.002** |
|  | **＞20** | **75** | **85** |  |  | **51** | **72** |  |
|  | **Missing** |  |  |  |  |  |  |  |
| **TP(g/L)** |  |  |  |  |  |  |  |  |
|  | **≤70** | **65** | **62** | **0.123** |  | **52** | **54** | **0.567** |
|  | **＞70** | **51** | **56** |  |  | **46** | **43** |  |
|  | **Missing** | **7** |  |  |  |  |  |  |
| **ALB(g/L)** |  |  |  |  |  |  |  |  |
|  | **≤40** | **49** | **30** | **0.876** |  | **36** | **40** | **0.357** |
|  | **＞40** | **67** | **88** |  |  | **61** | **59** |  |
|  | **Missing** | **6** |  |  |  |  |  |  |
| **ALT(U/L)** |  |  |  |  |  |  |  |  |
|  | **≤35** | **59** | **55** | **0.692** |  | **57** | **49** | **0.045** |
|  | **＞35** | **57** | **63** |  |  | **42** | **48** |  |
|  | **Missing** | **6** |  |  |  |  |  |  |
| **AST(U/L)** |  |  |  |  |  |  |  |  |
|  | **≤35** | **69** | **49** | **0.006** |  | **66** | **33** | **<0.001** |
|  | **＞35** | **47** | **69** |  |  | **33** | **64** |  |
|  | **Missing** | **6** |  |  |  |  |  |  |
| **GGT(U/L)** |  |  |  |  |  |  |  |  |
|  | **≤60** | **65** | **49** | **0.055** |  | **51** | **38** | **0.146** |
|  | **＞60** | **51** | **69** |  |  | **62** | **58** |  |
|  | **Missing** | **11** |  |  |  |  |  |  |
| **Tumor size(cm)** | |  |  |  |  |  |  |  |
|  | **≤5** | **83** | **44** | **<0.001** |  | **66** | **32** | **<0.001** |
|  | **＞5** | **35** | **74** |  |  | **24** | **58** |  |
|  | **Missing** | **20** |  |  |  |  |  |  |
| **Tumor number** | |  |  |  |  |  |  |  |
|  | **Single** | **92** | **89** | **0.644** |  | **75** | **72** | **0.048** |
|  | **Multiple** | **26** | **29** |  |  | **15** | **18** |  |
|  | **Missing** | **20** |  |  |  |  |  |  |
| **Tumor differentiation** | | |  |  |  |  |  |  |
|  | **Ⅰ-Ⅱ** | **30** | **20** | **0.111** |  | **19** | **9** | **0.005** |
|  | **Ⅲ-Ⅳ** | **87** | **97** |  |  | **63** | **81** |  |
|  | **Missing** | **30** |  |  |  |  |  |  |
| **Tumor encapsulation** | | |  |  |  |  |  |  |
|  | **Yes** | **90** | **95** | **<0.001** |  | **41** | **32** | **0.889** |
|  | **None** | **28** | **23** |  |  | **31** | **24** |  |
|  | **Missing** | **72** |  |  |  |  |  |  |
| **Satellite lesion** | |  |  |  |  |  |  |  |
|  | **Yes** | **9** | **19** | **0.044** |  | **2** | **3** | **0.653*^c^*** |
|  | **No** | **109** | **99** |  |  | **70** | **53** |  |
|  | **Missing** | **72** |  |  |  |  |  |  |
| **Vascular tumor thrombus** | | |  |  |  |  |  |  |
|  | **Yes** | **11** | **8** | **0.018** |  | **1** | **5** | **0.087 *^c^*** |
|  | **No** | **107** | **110** |  |  | **71** | **52** |  |
|  | **Missing** | **71** |  |  |  |  |  |  |
| **TNM stage** | |  |  |  |  |  |  |  |
|  | **Ⅰ** | **69** | **68** | **0.778** |  | **43** | **27** | **0.006** |
|  | **Ⅱ-Ⅳ** | **49** | **50** |  |  | **22** | **37** |  |
|  | **Missing** | **71** |  |  |  |  |  |  |

***^a^* HCC, hepatocellular carcinoma; DCP, des-gamma-carboxy prothrombin; AFP, alpha-fetoprotein; HBsAg, hepatitis B surface antigen; HBeAg, hepatitis B e antigen; TP, total protein; ALB, albumin; ALT, alanine aminotransferase; AST, aspartate transaminase; GGT, gamma-glutamyl transpeptidase.**

***^b^* Cutoff value of serum DCP for HCC diagnosis was 40 mAU/ml.**

***^c^* Fisher exact test. Chi-square tests for all the other analysis.**
