# Supplementary material for: Diagnostic Evaluation of Des-Gamma-Carboxy Prothrombin versus α-Fetoprotein for Hepatitis B Virus-Related Hepatocellular Carcinoma in China: A Large-Scale, Multicentre Study
Source: PLoS One. 2016 Apr 12;11(4):e0153227. doi: 10.1371/journal.pone.0153227 (PMC4829182; doi:10.1371/journal.pone.0153227)
Supplement: S4 Table — (DOC) [file pone.0153227.s007.doc]

S4 Table. Differences of clinical features between the DCP negative and positive groups

|  | **DCP negative** | **DCP positive** | ***P* value** |
| --- | --- | --- | --- |
|  | **n=24** | **n=117** |  |
| **Age, mean (range), y** | **54 (22-66)** | **49 (10-70)** | ***0.436*** |
| **Sex (male, %)** | **79** | **84** | ***0.206*** |
| **Positive for HBsAg (%)** | **83** | **86** | ***0.174*** |
| **TBIL (g/L)** | **12.35 (6.5, 41.9)** | **13.4 (4.6, 32.8)** | ***0.892*** |
| **ALB (g/L)** | **40.4 (35.5, 48.9)** | **41.6 (24.8, 53.3)** | ***0.445*** |
| **ALT (IU/L)** | **36.5 (12, 242)** | **34.5 (8, 1253)** | ***0.78*** |
| **AST (IU/L)** | **28.7 (17, 294)** | **35 (13, 1078)** | ***0.131*** |
| **AFP (ng/ml)** | **24.6 (1.8, 1210)** | **290 (1.6, 1210)** | ***0.037*** |
| **Tumor Size (cm)** | **3.05 (1.3, 19.2)** | **5 (1.4, 19.4)** | ***0.002*** |
| **Tumor Number (single, %)** | **87** | **81** | ***0.658*** |
| **TNM stage (TNM Ⅰ, %)** | **75** | **58** | ***0.007*** |
| **Data are expressed as median (range) or percentage. Abbreviations: DCP, des-gamma-carboxy prothrombin; HbsAg, hepatitis B e antigen; TBIL,** **total bilirubin; ALB, albumin; ALT, alanine aminotransferase; AST,** **aspartate transaminase; AFP, α-fetoprotein; TNM, tumor-nodes-metastasis.** | | | |
