# Supplementary material for: Diagnostic Evaluation of Des-Gamma-Carboxy Prothrombin versus α-Fetoprotein for Hepatitis B Virus-Related Hepatocellular Carcinoma in China: A Large-Scale, Multicentre Study
Source: PLoS One. 2016 Apr 12;11(4):e0153227. doi: 10.1371/journal.pone.0153227 (PMC4829182; doi:10.1371/journal.pone.0153227)
Supplement: S5 Table — (DOC) [file pone.0153227.s008.doc]

S5 Table. Univariate and multivariate analysis of risk factors affecting disease-free survival

|  | **Univariate Analysis** | | |  | **Multivariate Analysis** | | |
| --- | --- | --- | --- | --- | --- | --- | --- |
|  | **RR** | **95% CI** | ***P*** |  | **RR** | **95% CI** | ***P*** |
| **Age** | **1.003** | **0.975, 1033** | ***0.816*** |  | **—** | **—** | **NS** |
| **Sex (male)** | **1.934** | **0.692, 5.406** | ***0.209*** |  | **—** | **—** | **NS** |
| **Positive for HBsAg** | **0.623** | **0.245, 1.581** | ***0.623*** |  | **—** | **—** | **NS** |
| **AFP (>20 ng/ml)** | **1.064** | **0.548, 2.065** | ***0.855*** |  | **—** | **—** | **NS** |
| **DCP (>40 mAU/ml)** | **3.21** | **0.993,10.374** | ***0.051*** |  | **—** | **—** | **NS** |
| **ALT (IU/L)** | **0.999** | **0.995, 1.003** | ***0.621*** |  | **—** | **—** | **NS** |
| **Tumor Size** | **1.14** | **1.069, 1.216** | ***＜0.001*** |  | **1.114** | **1.054, 1.188** | ***0.001*** |
| **Tumor Number** | **1.561** | **0.771, 3.164** | ***0.216*** |  | **—** | **—** | **NS** |
| **TNM stage** | **2.603** | **1.432, 4.733** | ***0.002*** |  | **2.176** | **1.172, 4.038** | ***0.014*** |
| **Abbreviations: 95% CI, 95% confidence interval; HBV, hepatitis B virus; NS, not significant; RR, risk ration; TNM, tumor-nodes-metastasis.** | | | | | | | |
