# Supplementary material for: Diagnostic Evaluation of Des-Gamma-Carboxy Prothrombin versus α-Fetoprotein for Hepatitis B Virus-Related Hepatocellular Carcinoma in China: A Large-Scale, Multicentre Study
Source: PLoS One. 2016 Apr 12;11(4):e0153227. doi: 10.1371/journal.pone.0153227 (PMC4829182; doi:10.1371/journal.pone.0153227)
Supplement: S6 Table — (DOC) [file pone.0153227.s009.doc]

S6 Table. Univariate and multivariate analysis of risk factors affecting overall survival

|  | **Univariate Analysis** | | |  | **Multivariate Analysis** | | |
| --- | --- | --- | --- | --- | --- | --- | --- |
|  | **RR** | **95% CI** | ***P*** |  | **RR** | **95% CI** | ***P*** |
| **Age** | **0.992** | **0.956, 1.030** | ***0.682*** |  | **—** | **—** | **NS** |
| **Sex (male)** | **1.487** | **0.447, 4.955** | ***0.518*** |  | **—** | ***—*** | **NS** |
| **Positive for HBsAg** | **2.051** | **0.278, 15.142** | ***0.481*** |  | **—** | ***—*** | **NS** |
| **AFP ( >20 ng/ml)** | **1.275** | **0.512, 3.176** | ***0.602*** |  | **—** | ***—*** | **NS** |
| **DCP (>40 mAU/ml)** | **2.734** | **0.645, 11.584** | ***0.172*** |  | **—** | ***—*** | **NS** |
| **ALT** | **0.999** | **0.995, 1.004** | ***1.004*** |  | ***—*** | ***—*** | **NS** |
| **Tumor Size** | **1.158** | **1.073, 1.250** | ***＜0.001*** |  | **1.13** | **1.046, 1.22** | ***0.002*** |
| **Tumor Number** | **2.259** | **0.981, 5.200** | ***0.055*** |  | ***—*** | ***—*** | **NS** |
| **TNM stage** | **2.873** | **1.303, 6.335** | ***0.009*** |  | **2.286** | **0.005, 5.203** | ***0.049*** |
| **Abbreviations: 95% CI, 95% confidence interval; HBV, hepatitis B virus; NS, not significant; RR, risk ration; TNM, tumor-nodes-metastasis.** | | | | | | | |
