# Supplementary material for: Diagnostic Evaluation of Des-Gamma-Carboxy Prothrombin versus α-Fetoprotein for Hepatitis B Virus-Related Hepatocellular Carcinoma in China: A Large-Scale, Multicentre Study
Source: PLoS One. 2016 Apr 12;11(4):e0153227. doi: 10.1371/journal.pone.0153227 (PMC4829182; doi:10.1371/journal.pone.0153227)
Supplement: S7 Table — (DOC) [file pone.0153227.s010.doc]

S7 Table. The reproducibility of the assay

| **Level of**  **DCP** | **Intra-assay**  **Mean±SD** | **CV(%)** | **Inter-assay**  **Mean±SD** | **CV(%)** |
| --- | --- | --- | --- | --- |
| **Low** | **47.33±1.15** | **2.44** | **47.33±1.22** | **2.59** |
| **Middle** | **553.33±5.51** | **1.00** | **553.89±9.56** | **1.73** |
| **High** | **6406±35.03** | **0.55** | **6493.78±85.71** | **1.32** |
